# Toll-Like Receptors Represent an Important Link for Sex Differences in Cardiovascular Aging and Diseases

**DOI:** 10.3389/fragi.2021.709914

**Published:** 2021-06-24

**Authors:** Cinthya Echem, Eliana H. Akamine

**Affiliations:** Department of Pharmacology, Institute of Biomedical Sciences, University of São Paulo, São Paulo, Brazil

**Keywords:** toll-like receptor, sex hormone, sex difference, cardiovascular disease, cardiovascular aging

## Abstract

Human life span expectancy has increased, and aging affects the organism in several ways, leading, for example, to an increased risk of cardiovascular diseases. Age-adjusted prevalence of the cardiovascular diseases is higher in males than females. Aging also affects the gonadal sex hormones and the sex differences observed in cardiovascular diseases may be therefore impacted. Hormonal changes associated with aging may also affect the immune system and the immune response is sexually different. The immune system plays a role in the pathogenesis of cardiovascular diseases. In this context, toll-like receptors (TLRs) are a family of pattern recognition receptors of the immune system whose activation induces the synthesis of pro-inflammatory molecules. They are expressed throughout the cardiovascular system and their activation has been widely described in cardiovascular diseases. Some recent evidence demonstrates that there are sex differences associated with TLR responses and that these receptors may be affected by sex hormones and their receptors, suggesting that TLRs may contribute to the sex differences observed in cardiovascular diseases. Recent evidence also shows that sex differences of TLRs in cardiovascular system persists with aging, which may represent a new paradigm about the mechanisms that contribute to the sex differences in cardiovascular aging. Therefore, in this mini review we describe the latest findings regarding the sex differences of TLRs and associated signaling in cardiovascular diseases during aging.

## Introduction

Over the past years the world population is aging and living longer, which have increased the human lifespan expectancy. According to the World Health Organization, in the year of 2020 the number of adults over 60 years exceeded 1 billion people, which represents an increase of 2.5 times in relation to 1980. In addition, the prospect is that this amount of aged people will continue to grow and reach about 1.4 billion in 2030 and about 2.1 billion in 2050 (World Health Organization, 2020). It should be stressed, however, that the COVID-19 pandemic may have a large impact on mortality, both in terms of number of deaths and years of life lost, especially in older adults and men (Arolas et al., 2021).

Biological aging is a result of accumulated molecular and cellular damage, leading to disabilities and a greater risk of morbidity and mortality in the older adults, mainly due to the development of aging-associated diseases (Costa et al., 2015). In this context, the prevalence of cardiovascular diseases (CVDs) increases with age in males and females (American Heart Association, 2021), which represents the leading cause of death in the older adults (Centers for Disease Control and Prevention, 2020). There are several possible explanations for these enhanced risks of CVDs in aging, since the increased exposure time to the cardiovascular risk factors and progressive deterioration in structure and function of cardiovascular system over time make aging a strong risk factor for CVDs development, such as arterial hypertension, atherosclerosis, myocardial infarction and stroke in older persons (Lakatta and Levy, 2003; North and Sinclair, 2012). It should be stressed that chronic low-grade inflammation is a major hallmark of aging and plays a central role in aging-associated chronic diseases (Feldman et al., 2015).

The aging process also affects the gonadal sex hormones and although the senescence leads to a natural decline in sex hormones in both men and women, this reduction is more pronounced in women with the onset of menopause. This reduction in basal levels of sex hormones in aging directly impacts the sex differences observed in CVDs. In fact, women until mid-life have lower development and severity of CVDs compared to the same aged men; however, these differences tend to be equal or higher in post-menopausal women when compared to men (Moreau, 2019; American Heart Association, 2021).

Longitudinal studies demonstrate that menopause is associated with changes in lipid profile, weight gain, metabolic syndrome, epicardial and pericardial fat deposition, among others, which leads the menopause to be a risk for CVDs. In addition, the age of menopause onset is also an important factor for cardiovascular risk, as women with early-onset menopause have higher CVD risk than women with later-onset menopause (El Khoudary et al., 2020; Prabakaran et al., 2021).

Many reports demonstrate that sex hormones and their receptors can influence the cardiovascular health through complex pathways and the cardioprotective effects observed in pre-menopausal women are widely associated with estrogen benefits (Stanhewicz et al., 2018). In this regard, many studies suggest that hormone replacement therapy in menopausal women may have beneficial effects on the cardiovascular system. Furthermore, lifestyle interventions, with health diet, exercise and non-smoking, are fundamental to reach the cardiovascular health in menopausal women (Prabakaran et al., 2021).

Several studies show that sex hormones may also affect the immune system, since it is known that immune activation also shows sex differences. Indeed, women develop a stronger innate and adaptive immune responses when compared to men (Klein and Flanagan, 2016). Increasing evidence shows that the immune system activation has a significant role in the pathogenesis of CVDs (Ilatovskaya et al., 2019). Among the components of immune system that contribute to the pathogenesis of CVDs, the activation of toll-like receptors (TLRs) and their signaling pathway have been widely described as having a significant role in the development and progression of these diseases. Sex hormones may have an influence in the TLR signaling, which show sex differences.

In this mini review we will briefly summarize the latest findings regarding the sex differences of TLRs and associated signaling in cardiovascular aging and diseases, as well as we will highlight open points that need to be answered in future research.

### Toll-Like Receptors

The innate immune system is related to the initialization of inflammation, which involves TLRs. The TLRs are an important family of pattern recognition receptors present in immune and non-immune cells, being expressed throughout the cardiovascular system. Currently, thirteen subtypes of TLRs (TLR1-TLR13) were identified in mammals, of which eleven (TLR1-TLR11) are expressed in humans (Parizadeh et al., 2018; Dela Justina et al., 2020). These receptors can be activated by pathogen-associated molecular patterns (PAMPs) from virus, bacteria or fungi, as well as they can be activated by endogenous damage-associated molecular patterns (DAMPs), even in the absence of pathogens (Beutler, 2004). DAMPs include heat shock proteins, mitochondrial DNA, extracellular matrix fragments, uric acid and other factors (Goulopoulou et al., 2016).

TLR activation recruits adapter molecules, such as MyD88 (myeloid differentiation factor 88), MAL/TIRAP (MyD88-adapter-like/TIR-associated protein), TRIF (Toll-receptor-associated activator of interferon), TRAM (Toll-receptor-associated molecule) and SARM (sterile-α and heat/armadillo motif protein), leading to activation of a signaling pathway, including mitogen-activated protein kinases and inflammatory gene transcription factors, such as nuclear factor-κ B and interferon regulating factors, which results in synthesis of several pro-inflammatory molecules (Goulopoulou et al., 2016).

Activation of TLRs by host-derived molecules is also described as a possible link between immune system activation and CVDs, since it has already been demonstrated that in CVDs there is an increase in some endogenous molecules, that may act as DAMPs and activate the TLRs and their associated signaling pathway (Goulopoulou et al., 2016). In addition to the increased DAMPs that have been described in CVDs, it has been reported that TLR expression is increased in cardiovascular system. TLR4 expression is increased in mesenteric arteries and left ventricle of male hypertensive rats and the antagonism of these receptors improved several hypertension-associated alterations, such as hypercontractility of resistance arteries, vascular inflammation and left ventricle hypertrophy, showing that these receptors may be a promising target in CVDs (Bomfim et al., 2012; Bomfim et al., 2015; Echem et al., 2015).

DAMP-induced chronic inflammation plays a major role in aging-related diseases (Feldman et al., 2015). Moreover, changes in expression or activity of TLRs also contribute to aging-associated inflammation. Aortic smooth muscle cells from aged female mice showed higher TLR4 protein and mRNA expression compared to young female mice (Song et al., 2012). Furthermore, TLR2 and TLR4 protein expression, pro-inflammatory molecules and some DAMPs were higher in aged than in young male mice (Karuppagounder et al., 2016; Ao et al., 2017). In another study, it was observed that although aged male mice hearts did not show a higher TLR4 protein expression compared to young male mice, the TLR4 knockout improved aging-associated cardiac changes, suggesting that this receptor is a possible therapeutic target in aging (Wang et al., 2018).

TLR4 mRNA expression was higher than TLR2 mRNA expression in plaque debris from older adults, and acute coronary syndrome patients showed higher plaque TLR4 expression than patients with stable angina (Satoh et al., 2016), suggesting that increases in TLR4 expression may contribute to plaque instability during aging. In fact, aged female Apoe/TLR4 knockout mice showed less plaque instability, with reduced necrosis area, macrophage concentration, apoptosis and intraplaque hemorrhage (Doddapattar et al., 2018).

Some recent evidence has shown that there are sex differences associated with TLRs responses in the cardiovascular system, as well as that these receptors can be affected by sex hormones and their receptors, which could contribute to the sex differences observed in CVDs. Furthermore, some recent evidence also shows that these sex differences associated with TLRs in cardiovascular system persists with aging (Table 1), which may represent a new paradigm about the mechanisms that contribute to sex differences in cardiovascular aging and diseases.

**TABLE 1:** TLRs, sex differences and cardiovascular aging.

TLRs subtypes	Species	Material	Observation	Reference
TLR3-TLR7	Males and females SHR	Renal cortex and mesenteric arteries	Males: Higher TLR7 expression in renal cortex. Females: Higher TLR3, TLR5, TLR6 expression in renal cortex and TLR6 expression in mesenteric arteries	Cordeau et al. (2016)
TLR9	Young males and females SHR	Serum and aorta	Males: Higher serum mtDNA. Males: mtDNA induces larger aorta contraction and increased pro-inflammatory molecules. Females: mtDNA reduces aorta pro-inflammatory molecules and prevent oxidative stress	Rossouw et al. (2002)
TLR4	Young males and females Sprague Dawley rats with PAH	Plasma and lung	Males: Higher plasma HMGB1 and higher TLR4 expression in lung	Vickers et al. (2007)
TLR1-TLR10	Aging men and women	Platelets	Women: Higher expression of platelets TLRs. Men and women have different association of platelets TLRs and cardiovascular risk factors	Costa et al. (2019)
TLR1, TLR6 and TLR10	Aging men and women	Whole blood	TLRs polymorphisms were associated with healthy aging. The distribution of these polymorphisms differs between men and women	Dahm et al. (2012)
TLR4	Aging men and women with arterial hypertension	Peripheral blood leukcocytes	TLR4 polymorphism were associated with lower left ventricular mass and lower prevalence of left ventricular hypertrophy only in women	Li et al. (2014)
TLR9	Aging men and women with VTE	Whole blood	TLR9 polymorphism is associated with risk of VTE recurrence only in women	Markle and Fish, (2014)
TLR8	Aging men and women with ischemic stroke	Venous blood	TLR8 polymorphism is associated with susceptibility to ischemic stroke only in men	Norata et al. (2006)

TLRs, toll-like receptors; SHR, spontaneously hypertensive rats; mtDNA, mitochondrial DNA; PAH, pulmonary arterial hypertension; HMGB1, high-mobility group box 1; VTE, venous thromboembolism.

## Toll-Like Receptors and Sex Hormones

Sex hormones may exert an influence on the expression and function of TLRs, since the TLR activation varies according to the hormonal fluctuation during menstrual cycle. In peripheral blood cells of young women, it was observed that the TLR responsiveness (except for TLR3, TLR7, and TLR9) varies throughout the menstrual cycle, with a greater production of pro-inflammatory cytokines in early luteal phase and a lower production in late luteal phase (Dennison et al., 2012). Besides that, it was also demonstrated that the production of pro-inflammatory cytokines by TLR7/8 and TLR9 activation, in spleen and dendritic cells, is dependent of ERα binding to the estrogen-responsive element and occurs independently of the estrogen presence (Cunningham et al., 2014). This reveals that estrogen receptors can activate the TLR signaling even in the absence of female sex hormones.

On the other hand, despite the fact that estrogen or estrogen receptor signaling appears to modulate the activation of TLR signaling, in the blood vessels, estrogen seems to have a different role, especially under injury conditions. For example, in human brain vascular smooth muscle cells, angiotensin II incubation induces several cellular damages, including modification of vascular smooth muscle cell phenotype and increased TLR4 and monocyte chemoattractant protein-1 expression. However, these effects were inhibited in the presence of estrogen (Xia et al., 2020).

In an animal model of preeclampsia, the treatment with estrogen was also associated with beneficial responses, such as anti-inflammatory, antioxidant and anti-vascular dysfunction effects in placenta, which were partly mediated by suppression of TLR4 and its downstream signaling (Lin et al., 2020). Moreover, in peripheral blood mononuclear cells from patients with preeclampsia, progesterone also suppresses the TLR4 and its downstream signaling in a dose-dependent manner (Zhu et al., 2013), showing that the cardioprotective effects of estrogens under injury may be, at least in part, associated with a downregulation of TLRs.

It should be expected, therefore, that the lack of female sex hormones can also modulate the TLRs, a fact that may have an important impact on the cardiovascular system of aging women. It was observed that ovariectomy induced an increased TLR2 protein expression in female brain. Furthermore, after cerebral ischemia and reperfusion, this menopausal model showed larger cerebral activity of TLR2, ischemic injury area and expression of apoptosis markers, a fact that was also associated with the estrogen receptor ERα. Therefore, it was suggested that the natural reduction of sex hormones or loss of ERα function with aging can lead to a deregulation of postischemic inflammation and increase the injury damage risk in brain of older women (Cordeau et al., 2016).

With aging and the onset of menopause, many women initiate hormone replacement therapies to combat the climacteric symptoms. Despite the fact that estrogen promotes a cardiovascular system protection in pre-menopausal women, even suppressing TLR signaling, some randomized studies suggest that hormone replacement therapy can lead to greater cardiovascular risk (Rossouw et al., 2002; Vickers et al., 2007). An explanation is that the type of hormone or doses used in the hormone replacement therapies may negatively affect the cardiovascular system in both direct and indirect ways (Rossouw et al., 2002; Vickers et al., 2007). Another hypothesis is that a late onset of estrogen therapy is detrimental for the cardiovascular system, since estrogen loses its beneficial action and becomes detrimental in blood vessels from aging females (Costa et al., 2019).

Although the underlying mechanisms are unknown, regulation of the immune system-related genes (including those coding for TLRs) by hormone replacement therapies may be involved in their effects of increasing the risk for CVDs. In menopausal women that received different types of hormone replacement therapy it was observed that treatments with a conventional dose of 17β-estradiol/norethisterone acetate or tibolone increased the TLR2 gene expression in blood cells, an effect that was not observed with a low dose of 17β-estradiol/ norethisterone acetate or raloxifene (Dahm et al., 2012). In human monocyte THP-1 cells, the incubation with 17β-estradiol also increased the TLR2 mRNA and protein expression in a dose-dependent manner, an event that occurred by means of the estrogen receptor ERα through the estrogen-responsive element (Li et al., 2014). This evidence suggests that the different modulation of TLRs by different types and doses of female sex hormones may contribute in part to the larger cardiovascular risk of some hormone replacement therapies in aging women.

Different from estrogen, testosterone generally promotes an immunosuppression effect (Markle and Fish, 2014). In the cardiovascular system, the male sex hormones can also affect the TLRs; however, the findings present in literature are few and conflicting. In human umbilical vein endothelial cells, it was described that the stimulus with lipopolysaccharide (TLR4 agonist) or with proinflammatory cytokine tumor necrosis factor-α increased the mRNA of inflammatory molecules, including TLR4. Despite that, these increases were reduced with dihydrotestosterone, a fact in part associated with androgen receptor AR (Norata et al., 2006). On the other hand, the opposite result was also described in human umbilical vein endothelial cells, since dihydrotestosterone exacerbated the increased protein expression of TLR4 and inflammatory molecules induced by lipopolysaccharide, an effect that was not associated with AR (Wan et al., 2013). The different doses and stimulus durations used in these studies may in part explain these opposite results. Moreover, with this evidence it is possible to suggest that the effects of male sex hormones on TLRs signaling may be influenced by different hormonal concentrations.

## Toll-Like Receptors, Sex Differences and Cardiovascular Aging

The evidence described above shows that TLRs are affected by sex hormones and their receptors, indicating that the TLRs activation can be different between males and females. In fact, scarce evidence shows that there are sex differences in TLRs responses in cardiovascular system and target-organ, which could remain during aging. Thus, the sex difference in TLRs could contribute to the sex differences observed in cardiovascular aging and diseases.

In spontaneously hypertensive rats (SHR), it was observed that renal cortex of kidney from males showed greater expression of 8 immune response-related genes when compared to females, including TLR7, whereas 25 genes, including TLR3, TLR5 and TLR6, were more expressed in females than in males. Similarly, it was also observed that mesenteric arteries from males showed a larger expression of 9 immune response-related genes when compared to females, whereas 17 genes were more expressed in females when compared to males, including TLR6 (Tipton and Sullivan, 2014). Therefore, these data show the need for further studies to clarify the role of each type of TLRs in the pathophysiology of CVDs in males and females in order to understand whether sex differences in TLRs contribute to the sex difference in cardiovascular aging.

Beyond the different expression between males and females, the activation of TLRs may also be different in both sexes due to the differentiated release of DAMPs in CVDs. For example, male SHR showed a higher serum mitochondrial DNA (TLR9 DAMP) when compared to females (Echem et al., 2019); likewise, in an animal model of pulmonary arterial hypertension, males showed a higher circulating HMGB1 (TLR4 DAMP) when compared to females. In addition, in this animal model of pulmonary arterial hypertension, males also showed higher TLR4 protein expression in lungs when compared to females, suggesting that the larger activation of the HMGB1/TLR4 axis may contribute to the worse outcomes of pulmonary arterial hypertension observed in males (Zemskova et al., 2020).

It is important to highlight that it has already been described that the DAMPs released in CVDs can induce different responses of the cardiovascular system in males and females. In male SHR, incubation of aorta with mitochondrial DNA increased the contractile response induced by phenylephrine, as well as increased the release of pro-inflammatory cytokines. However, in female SHR, the same aorta incubation with mitochondrial DNA did not affect the contractile response induced by phenylephrine, as well as reduced the release of pro-inflammatory molecules and prevented the phenylephrine-induced oxidative stress (Echem et al., 2019).

These sex differences associated with TLRs in cardiovascular system seem to persist with aging and differently impact the cardiovascular health of aged men and women. In fact, in a study with aging subjects, the expression of TLRs in platelets and its association with different cardiovascular risk factors varied according to sex. It was observed that platelets of aging women have a higher expression of TLRs when compared to platelets of aging men. In addition, cardiovascular risk factors such as body mass index, total cholesterol to high density lipoprotein ratio and increased prothrombotic markers are associated with a higher expression of TLRs in aging women. However, blood pressure, hypertensive treatment, lipid treatment and increased inflammatory markers are associated with a larger expression of TLRs in aging men (Koupenova et al., 2015).

The TLR polymorphisms have also been associated with the sex differences of CVDs in older adults, and these polymorphisms have already been described as having a protective or deleterious role on cardiovascular system. For example, the presence of TLR1, TLR6 and, TLR10 polymorphisms in whole blood of aged men and women were associated with a healthy aging. In addition, it was observed that the distribution of these polymorphisms varied according to the sex (Hamann et al., 2015). Sex differences associated with TLR4 polymorphism in aging have also been described. In hypertensive subjects with over 55 years of age, it was observed that women carrying the TLR4 Asp299Gly polymorphism in peripheral blood leukcocytes showed lower left ventricular mass and lower prevalence of left ventricular hypertrophy; however, the presence of this polymorphism in men did not affect the ventricular structure (Sales et al., 2010).

Furthermore, sex differences associated with TLR9 and TLR8 polymorphisms have also been described in CVDs. In whole blood of aging patients with venous thromboembolism, it was observed an association between the TLR9 rs5743836 polymorphism and the risk of venous thromboembolism recurrence in aged women; however, this association was not observed in aged men (Ahmad et al., 2017). Moreover, in whole blood of aging patients with ischemic stroke, it was observed that the TLR8 rs3764880 polymorphism is associated with the susceptibility to ischemic stroke in aging men. However, this association was not observed in aged women (Gu et al., 2016). This evidence shows that different types of polymorphisms can affect the aged cardiovascular system in different ways, as well as these polymorphisms can also differently affect the cardiovascular system of aged men and women.

## Conclusion and Future Perspectives

**FIGURE 1:**
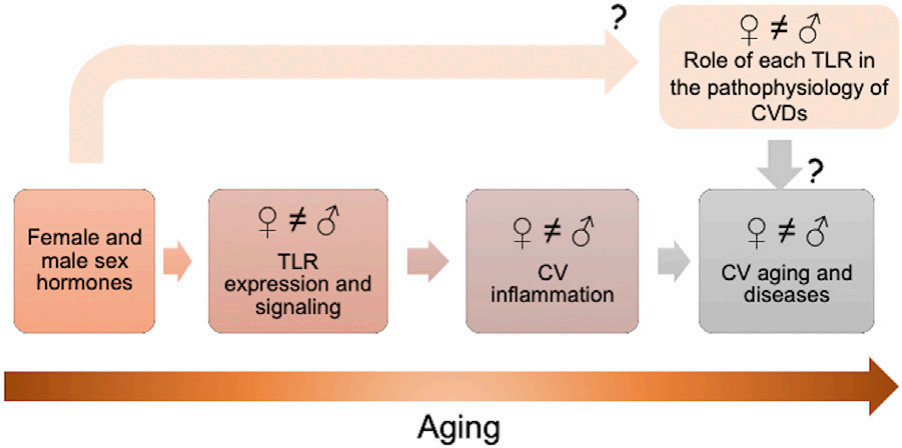
Sex hormones and their receptors can affect the toll-like receptors (TLRs) expression and signaling. TLRs act differently in cardiovascular (CV) system and cardiovascular diseases (CVDs) of older men and women. Further studies are needed to comprehend the real impact of male and female sex hormones on TLR signaling in CV system and the role of each TLR in the pathophysiology of CVDs in males and females.

In this present mini review, we summarize the latest evidence that shows that sex hormones and their receptors can affect the TLR expression and signaling. In addition, we also described the latest evidence that shows that TLRs act differently in cardiovascular system and cardiovascular diseases of men and women, a fact that is also observed during aging (Figure 1).

However, many questions are still open and further studies are needed to comprehend the real impact of male and female sex hormones on TLR signaling in cardiovascular system. Moreover, more studies that evaluate older adults of both sexes and the role of each TLR in the pathophysiology of CVDs are also needed to understand the real contribution of TLRs in the pathophysiology of CVDs in males and females.

Finally, we suggest that the TLRs might contribute to the sex differences in CVDs. Although the evidence that supports this hypothesis appears still limited, further studies will certainly enlighten the issue.

## Abbreviations

CVDs, cardiovascular diseases; DAMPs, damage-associated molecular patterns; PAMPs, pathogen-associated molecular patterns; SHR, spontaneously hypertensive rats; TLRs, toll-like receptors.
